# Proteomic and genomic profiling of pancreatic cancer

**DOI:** 10.1007/s10565-019-09465-9

**Published:** 2019-02-15

**Authors:** Daniel Ansari, William Torén, Qimin Zhou, Dingyuan Hu, Roland Andersson

**Affiliations:** 1 Department of Surgery, Clinical Sciences Lund, Skåne University Hospital, Lund University, SE-221 85 Lund, Sweden; 2 School of Ophthalmology and Optometry, Eye Hospital, Wenzhou Medical University, Wenzhou, China; 3 Department of Gastroenterology, The Second Affiliated Hospital and Yuying Children’s Hospital of Wenzhou Medical University, Wenzhou, China

**Keywords:** Pancreatic cancer, Proteomics, Genomics, Proteogenomics, Biomarkers

## Abstract

Pancreatic cancer remains the most fatal human tumor type. The aggressive tumor biology coupled with the lack of early detection strategies and effective treatment are major reasons for the poor survival rate. Collaborative research efforts have been devoted to understand pancreatic cancer at the molecular level. Large-scale genomic studies have generated important insights into the genetic drivers of pancreatic cancer. In the post-genomic era, protein sequencing of tumor tissue, cell lines, pancreatic juice, and blood from patients with pancreatic cancer has provided a fundament for the development of new diagnostic and prognostic biomarkers. The integration of mass spectrometry and genomic sequencing strategies may help characterize protein identities and post-translational modifications that relate to a specific mutation. Consequently, proteomic and genomic techniques have become a compulsory requirement in modern medicine and health care. These types of proteogenomic studies may usher in a new era of precision diagnostics and treatment in patients with pancreatic cancer.

## Introduction

Of all major human cancers, pancreatic ductal adenocarcinoma has the most dismal prognosis. Pancreatic ductal adenocarcinoma, henceforth referred to as pancreatic cancer, has risen to become the third leading cause of cancer death in the USA, with a 5-year survival rate of 8% (Siegel et al., 2018). Without scientific advances and clinical innovations, pancreatic cancer will become the second cancer killer within the coming years, after lung cancer (Rahib et al., 2014). Surgery is the only potentially curative treatment available today. Unfortunately, only about 15–20% of patients have localized tumors that can be surgically resected (Ansari et al., 2016; Yeo and Cameron, 1999). Recent advances in pancreatic surgery, including centralization to high-volume centers, have increased the resection rates and decreased short-term mortality (Ahola et al., 2017). However, the long-term survival for pancreatic cancer has only minimally improved in the past six decades, when all tumor stages are considered (Ansari et al., 2016).

The diagnosis of pancreatic cancer is often delayed due to lack of early, disease-specific symptoms. Furthermore, there is a deficiency of accurate molecular strategies for diagnosis, prognosis, and treatment selection. Carbohydrate antigen 19-9 (CA 19-9) is the only clinical biomarker for pancreatic cancer that is approved by the FDA. Measurement of CA 19-9 in serum can be used for diagnostic workup of pancreatic lesions or for evaluation of treatment response after surgery or chemotherapy (Duffy et al., 2010). However, CA 19-9 is not recommended in a screening setting, due to inadequate sensitivity and specificity.

The progress within proteomic and genomic research has greatly increased our knowledge regarding the molecular basis of pancreatic cancer (Ansari et al., 2014; Bailey et al., 2016). Translating these proteomic and genomic findings into clinical practice is the next phase in order to provide innovations that can improve clinical management. New biomarkers represent an unmet need in pancreatic cancer. Protein or genomic markers detected in body fluids can be used for early diagnosis, prognosis, prediction of treatment response, and development of new targeted treatment. This review highlights the expanding importance of proteomic and genomic research in pancreatic cancer.

## CA 19-9

CA 19-9 is an oligosaccharide that is expressed by mucins. The sensitivity of CA 19-9 for diagnosis of pancreatic cancer is estimated at 79%, with a specificity at 83% (Poruk et al., 2013). Increased levels of CA 19-9 can be caused by other pancreaticobiliary diseases, such as chronic pancreatitis or biliary obstruction. Approximately 10% of the population cannot express CA 19-9, due to the lack of the necessary Lewis glycosyltransferase (Goonetilleke and Siriwardena, 2007; Kawai et al., 2008; Kim et al., 2004).

CA 19-9 is often used together with CT and MRI in the diagnostic workup for pancreatic cancer. However, CA 19-9 is not accepted as a screening test for pancreatic cancer (Locker et al., 2006). Increased CA 19-9 levels may indicate disease progression, recurrence and poor outcome in patients with pancreatic cancer (Duffy et al., 2010).

In spite of its limitations, CA 19-9 is still the most widely used tumor marker for pancreatic cancer. Proteomic and genomic studies must therefore use CA 19-9 as a benchmark for which to compare, evaluate, and develop new biomarkers.

## Proteomic biomarkers

Mass spectrometry (MS) is a central technological platform in proteomic research. Biological samples are complex and usually require that the proteome be separated prior to analysis. Separations based on gel chromatography or liquid chromatography are commonly performed. MS analysis of proteins is done either as intact proteins (top–down) or as enzymatically digested protein peptides (bottom–up). Several methods have been developed for protein quantification, such as chemical labeling by isobaric tags for relative and absolute quantitation (iTRAQ) or isotope-coded affinity tag (ICAT), metabolic labeling by SILAC, or label-free techniques. Antibody techniques such as immunohistochemistry and ELISA are also important tools in proteomic research once the target protein has been established.

Tumor-derived proteins detectable in tumor tissue, cell lines, pancreatic juice, and blood have great possibility to be used as tumor biomarkers. Such proteins can hopefully be applied for improving clinical management (Tables 1, 2, 3, and 4).

**Table 1:** Selected proteomic biomarkers in tissue

Biomarker	Method of detection	Application	Reference
Actinin-4	iTRAQ, ICAT, LC-MS/MS, IHC	Diagnostic	(Pan et al., 2009)
Annexin A2	ICAT, LC-MS/MS	Diagnostic	(Chen et al., 2007a)
Bcl-2	IHC	Prognostic	(Nio et al., 2001)
	IHC	Prognostic	(Dong et al., 2005)
Cathepsin D	ICAT, LC-MS/MS	Diagnostic	(Chen et al., 2007a)
CD34	IHC	Prognostic	(Ikeda et al., 1999)
	IHC	Prognostic	(Fujioka et al., 2001)
CEACAM5	LC-MS/MS	Diagnostic	(Pan et al., 2014)
COX-2	IHC	Prognostic	(Juuti et al., 2006)
	IHC	Prognostic	(Matsubayashi et al., 2007)
Galectin-1	iTRAQ, ICAT, LC-MS/MS, IHC	Diagnostic	(Pan et al., 2009)
H1.3	LC-MS/MS	Prognostic	(Bauden et al., 2017)
hENT1	IHC	Predictive/prognostic	(Greenhalf et al., 2014)
	IHC	Predictive/prognostic	(Farrell et al., 2009)
	IHC	Predictive/prognostic	(Marechal et al., 2009)
IGFBP2	ICAT, LC-MS/MS	Diagnostic	(Chen et al., 2007a)
IGFBP3	LC-MS/MS	Diagnostic	(Pan et al., 2014)
Integrin 1	ICAT, LC-MS/MS	Diagnostic	(Chen et al., 2007a)
Ki-67	IHC	Prognostic	(Linder et al., 1997)
	IHC	Prognostic	(Shyr et al., 1999)
	IHC	Prognostic	(Yamamoto et al., 2004)
	IHC	Prognostic	(Karamitopoulou et al., 2010)
Laminin beta 1	iTRAQ, ICAT, LC-MS/MS, IHC	Diagnostic	(Pan et al., 2009)
LGALS3BP	LC-MS/MS	Diagnostic	(Pan et al., 2014)
MUC5AC	LC-MS/MS	Diagnostic	(Pan et al., 2014)
P27	IHC	Prognostic	(Lu et al., 2000)
	IHC	Prognostic	(Juuti et al., 2003)
	IHC	Prognostic	(Fukumoto et al., 2004)
P53	IHC	Prognostic	(Linder et al., 1997)
	IHC	Prognostic	(Ahrendt et al., 2000)
Plasminogen	ICAT, LC-MS/MS	Diagnostic	(Chen et al., 2007a)
S100A4	IHC	Prognostic	(Ai et al., 2008)
	IHC	Prognostic	(Oida et al., 2006)
Survivin	IHC	Prognostic	(Kami et al., 2004)
	IHC	Prognostic	(Tonini et al., 2005)
TGF-β1	IHC	Prognostic	(Nio et al., 2005)
	IHC	Prognostic	(Hashimoto et al., 2001)
VEGF	IHC	Prognostic	(Ikeda et al., 1999)
	IHC	Prognostic	(Sun et al., 2007)
	IHC	Prognostic	(Ai et al., 2008)

*ICAT* isotope-coded affinity tag, *IHC* immunohistochemistry, *iTRAQ* isobaric tags for relative and absolute quantitation, *LC-MS/MS* liquid chromatography tandem mass spectrometry

**Table 2:** Selected proteomic biomarkers in cell lines

Biomarker	Method of detection	Reference
ApoE	SILAC, LC-MS/MS, IHC	(Gronborg et al., 2006)
CD9	SILAC, LC-MS/MS, IHC	(Gronborg et al., 2006)
Fibronectin receptor	SILAC, LC-MS/MS, IHC	(Gronborg et al., 2006)
Perlecan	SILAC, LC-MS/MS, IHC	(Gronborg et al., 2006)
S100A6	Laser capture microdissection, 2-DE, IHC	(Shekouh et al., 2003)
S100A8	Laser capture microdissection, 2-DE	(Sheikh et al., 2007)
S100A9	Laser capture microdissection, 2-DE	(Sheikh et al., 2007)
SDF4	SILAC, LC-MS/MS, IHC	(Gronborg et al., 2006)
SMAD4	IHC	(Wang et al., 2017)

*2-DE* two-dimensional gel electrophoresis, *IHC* immunohistochemistry, *LC-MS/MS* liquid chromatography tandem mass spectrometry, *SILAC* stable isotope labeling with amino acids in cell culture

**Table 3:** Selected proteomic biomarkers in pancreatic juice

Biomarker	Method of detection	Reference
A1BG	DIGE, LC-MS/MS, IHC, Western blot	(Tian et al., 2008)
Caldecrin	ICAT, LC-MS/MS	(Chen et al., 2007b)
DJ-1	DIGE, LC-MS/MS, IHC, Western blot	(Tian et al., 2008)
FGB	ICAT, LC-MS/MS	(Chen et al., 2007b)
Lithostathine I α	2-DE, MALDI-TOF-MS	(Zhou et al., 2007)
MMP-9	DIGE, LC-MS/MS, IHC, Western blot, ELISA	(Tian et al., 2008)
L1CAM	ICAT, LC-MS/MS	(Chen et al., 2007b)
Plasminogen	ICAT, LC-MS/MS	(Chen et al., 2007b)
S100A8	GeLC-MS/MS	(Chen et al., 2014)
S100A9	GeLC-MS/MS	(Chen et al., 2014)

*2-DE* two-dimensional gel electrophoresis, *DIGE* difference gel electrophoresis, *ELISA* enzyme-linked immunosorbent assay, *GeLC-MS/MS* gel-enhanced liquid chromatography tandem mass spectrometry, *ICAT* isotope-coded affinity tag, *IHC* immunohistochemistry, *LC-MS/MS* liquid chromatography tandem mass spectrometry, *MALDI-TOF-MS* matrix assisted laser desorption ionization-time of flight mass spectrometry

**Table 4:** Selected proteomic biomarkers in serum/plasma

Biomarker	Method of detection	Sensitivity (%)	Specificity (%)	Reference
CA 19-9	ELISA	78	83	(Poruk et al., 2013)
DKK1	ELISA	89	79	(Han et al., 2015)
Exosomal glypican-1	UPLC-MS	100	100	(Melo et al., 2015)
HSP-27	SELDI, ELISA	100	84	(Melle et al., 2007)
IL-11	ELISA	98	70	(Ren et al., 2014)
MIC-1	ELISA	66	96	(Wang et al., 2014)
Xylitol + 1,5-anhydro-D-glucitol + histidine + inositol	GC/MS	71–86	78–88	(Kobayashi et al., 2013)
CA 19-9 + MUC5AC	ELISA	75	83	(Kaur et al., 2017)
CA 19-9 + CA 242	ELISA	89	75	(Zhang et al., 2015)
CA 19-9 + IGF-1 + albumin	ELISA	94	95	(Ferri et al., 2016)
CA 19-9 + CEA + CA 125 + CA 242	ELISA	90	94	(Gu et al., 2015)
CA 19-9 + 5MC + H2AZ + H2A1.1 + H3K4Me2	ELISA	92	90	(Bauden et al., 2015)
CA 19-9 + CEA + HGF + OPN + ctDNA	Luminex bead-based immunoassay, PCR	64	99.5	(Cohen et al., 2017)
CA 19-9 + THBS2	ELISA	87	98	(Kim et al., 2017)
29-Biomarker panel	Antibody microarray	94	95	(Mellby et al., 2018)

*ELISA* enzyme-linked immunosorbent assay, *GC/MS* gas chromatography/mass spectrometry, *PCR* polymerase chain reaction, *SELDI* surface-enhanced laser desorption/ionization, *UPLC-MS* ultra performance liquid chromatography mass spectrometry

### Tissue

Pancreatic cancer tissue is the most direct source of tumor-associated proteins. With improvements in proteomic technology, it has become possible to analyze the pancreatic cancer proteome with impressive depth and detail, also describing post-translational modifications.

Detection and characterization of precursor lesions can enable new insights into early diagnosis and timely treatment of pancreatic cancer. Pancreatic intraepithelial neoplasia-3 (PanIN-3) is an established precursor lesion of pancreatic cancer. Quantitative MS analysis using iTRAQ and ICAT was applied to study protein expression in PanIN-3, pancreatic cancer, and control tissues (Pan et al., 2009). The study found multiple aberrantly regulated proteins already in the earliest stages of pancreatic cancer development, as evident by the overlap of proteins in PanIN-3 and pancreatic cancer. Of the aberrantly regulated proteins in PanIN-3 compared to normal pancreas, multiple proteins could be categorized as being involved in cell motility, cell cycle regulation, and inflammation. Immunohistochemistry was conducted on chosen biomarker candidates. Galectin-1 and laminin beta-1 were overexpressed in the stroma adjacent to PanIN 3, while actinin-4 was overexpressed in the stroma and ductal epithelium of pancreatic cancer.

Another quantitative proteomic study investigated differences in protein expression between chronic pancreatitis and pancreatic cancer using ICAT (Chen et al., 2007a). Among the aberrantly expressed proteins in chronic pancreatitis, 40% were also altered in pancreatic cancer. The observations were further confirmed by Western blot and immunohistochemistry. Annexin A2 and IGFBP2 were found to be overexpressed in pancreatic cancer, but not in chronic pancreatitis. Integrin 1, cathepsin D, and plasminogen were overexpressed in both pancreatic cancer and chronic pancreatitis. The partly, mutual molecular signatures between chronic pancreatitis and pancreatic cancer have also been suggested by another study using label-free quantitative MS (Pan et al., 2011). These similarities in molecular expression indicate that inflammation has a central role in pancreatic cancer pathophysiology.

Post-translational modifications regulate the activity of most proteins. By studying these modifications, we can gain important insights into biological function and also improve biomarker discovery. One study compared the level of N-glycosylation of glycoproteins in pancreatic cancer and normal pancreatic tissue (Pan et al., 2014). Altered N-glycosylation in pancreatic cancer tissue compared to normal pancreatic tissue was reported for MUC5AC, LGALS3BP, CEACAM5, and IGFBP3. Another study displayed the increased level of N-glycosylation of Thy-1 membrane glycoprotein in pancreatic cancer (Foygel et al., 2013). In a previous study, we analyzed histone variants and their related post-translational modifications in patients with pancreatic cancer (Bauden et al., 2017). Using liquid chromatography-tandem mass spectrometry (LC-MS/MS), we found histone variant H1.3 to be differentially expressed in pancreatic cancer tissue as compared to normal controls. Histone variant H1.3 was found to be an independent marker of poor survival in patients undergoing surgical resection.

Protein markers can also be associated with disease course. In a previous study, we provided a systematic overview of immunohistochemical markers for survival (Ansari et al., 2011). Many independent prognostic markers were found, but only a limited number of markers were validated in an external study, including Ki-67, p27, p53, VEGF, Bcl-2, TGF-β1, survivin, COX-2, hENT1, CD34, and S100A4. Further validation steps are necessary before these markers can be used in clinical routine.

### Cell lines

Cell lines are a useful source of biomarkers, as they are easily accessible and enable proteomic analysis of secreted proteins. However, data interpretation is challenging as isolated cells may not be fully representative of patient tumors (Tonack et al., 2009). Several biomarkers have been derived from proteomic research in cell lines.

The secretome of pancreatic cancer cells has been analyzed using SILAC MS profiling (Gronborg et al., 2006). Multiple novel pancreatic cancer biomarkers were identified, including SDF4, perlecan, CD9, the fibronectin receptor and apoE. The proteins were additionally validated with Western blot and immunohistochemistry. Another study identified S100A6 as a pancreatic cancer biomarker in cell lines, using laser capture microdissection, two-dimensional gel electrophoresis (2-DE), MS, and immunohistochemistry (Shekouh et al., 2003).

Cell lines have also been used to study biomarkers for metastatic disease. Primary and metastatic cell lines of pancreatic cancer were analyzed by SDS-PAGE, in-gel tryptic digestion and LC-MS/MS. Only about half of identified proteins could be found in both cell types, indicating a remarkably different protein profile. Among the differentially expressed proteins were collagens, integrins, galectins, and cadherins that are functionally related to cell motility and adhesion (Liu et al., 2010). Another study compared cells from primary tumors and lymph node metastasis with laser capture microdissection in combination with LC-MS/MS. S100P and 14-3-3 sigma were validated as markers of lymphatic engagement using immunohistochemistry (Naidoo et al., 2012). These findings are clinically important, as metastasis is the most important cause of death in pancreatic cancer patients.

The stromal compartment is a major determinant in the biology of pancreatic cancer. Pancreatic stromal cells have been evaluated using laser capture microdissection in combination with 2-DE (Sheikh et al., 2007). S100A8 and S100A9 were found to be overexpressed in the tumor stroma and were also correlated to SMAD4 expression. Protein expression was primarily present on immune cells in the stroma.

### Pancreatic juice

Pancreatic juice is protein-rich and directly secreted from pancreatic ductal cells, making it a valuable source for proteomic studies. Samples can be collected from the pancreatic duct with endoscopic techniques such as ERCP (Tryliskyy and Bryce, 2018). Thus, pancreatic juice has an exceptional composition for proteomic studies but is less available than blood due to the invasive procedure to procure the samples.

Proteomic technologies have been applied to pancreatic juice to develop new biomarkers. Pancreatic juice from patients with pancreatic cancer, benign pancreatic disease, and gallstone disease was analyzed with 2-DE and MALDI-TOF-MS (Zhou et al., 2007). The study found that gallstone disease affects the protein composition of the pancreatic juice. Five proteins were found to be have decreased expression in pancreatic cancer, including lithostathine I α. Quantitative proteomic approaches have also been studied in the identification of biomarkers from pancreatic juice in the differentiation between pancreatic cancer and pancreatitis using ICAT and LC-MS/MS (Chen et al., 2007b). Plasminogen, fibrinogen β-chain, caldecrin, and neural cell adhesion molecule L1 were found to be upregulated. Another study used gel electrophoresis and LC-MS/MS (Tian et al., 2008). DJ-1, MMP-9, and A1BG were found to be upregulated in pancreatic juice and were further investigated by Western blot and immunohistochemistry.

Other mass spectrometric studies of pancreatic juice have found CEA, S100A6, S100A8, S100A9, and S100P to be differentially regulated between pancreatic cancer and benign conditions (Chen et al., 2014; Mori et al., 2013; Ohuchida et al., 2005).

### Serum

The use of a simple blood test to detect pancreatic cancer is an attractive strategy to improve early detection. Thus, much effort has been focused on the identification of serum markers for pancreatic cancer (Han et al., 2015; Melle et al., 2007; Ren et al., 2014). The identification of new biomarkers is, however, complicated by the low concentrations of biomarker candidates in blood and the presence of transport proteins, such as albumin, in high concentrations.

Metabolic disorders such as obesity and diabetes mellitus type II are known risk factors for pancreatic cancer. The pathophysiological mechanisms are not well recognized. It is speculated that several proteins associated with obesity and diabetes may also promote pancreatic tumor progression, such as adiponectin, leptin, glucose metabolic enzymes, MMP9, and VNN1 (Pothuraju et al., 2018). Furthermore, proteomic analyses based on MS have reported metabolites and metabolic enzymes as candidate blood biomarkers for diagnosis of pancreatic cancer. The markers histidine, inositol, xylitol, and 1,5-anhydro-d-glucitol were found to be outperform CA 19-9 in the diagnosis of pancreatic cancer from controls (Kobayashi et al., 2013).

Many studies have investigated the combinatory precision of CA 19-9 and various other biomarkers, in order to improve the diagnostic accuracy. For example, CA 19-9 coupled with MUC5AC provided a sensitivity of 75% and a specificity of 83% (Kaur et al., 2017). A meta-analysis reported that the sensitivity of CA 19-9 was increased to 89% when used together with CA 242 (Zhang et al., 2015). Another study found that a panel of serum CA 19-9, CA 125, CA 242, and CEA had a sensitivity and specificity of 90% and 94% (Gu et al., 2015). When using CA 19-9 as a diagnostic marker in combination with albumin and IGF-1, a sensitivity of 94% and a specificity of 95% were achieved (Ferri et al., 2016).

In a previous study, we used a novel immunoassay to profile circulating nucleosomes in patients with pancreatic cancer and controls (Bauden et al., 2015). A five-marker nucleosomes panel was found to be better than CA 19-9 in separating pancreatic cancer sera from healthy control sera, including H3K4Me2, H2AZ, 5MC, H2A1.1, and H2AK119Ub. The four nucleosomes H3K4Me2, H2AZ, 5MC, and H2A1.1 together with CA 19-9 yielded an AUC of 0.98, at a sensitivity of 92% with 90% specificity.

Recently, it was reported that CA 19-9 together with THBS2 may enhance the diagnosis of early-stage pancreatic cancer, when validated in large and independent patient groups (Kim et al., 2017). The CA 19-9 and THBS2 panel yielded a sensitivity of 87% with a specificity of 98%.

Antibody array technology has been applied to develop serum biomarker signatures for diagnosis of pancreatic cancer (Wingren et al., 2012). In a follow-up study, the serum panel was refined to include 29 proteins, which gave an AUC of 0.96 for stage I and II pancreatic cancers, with a sensitivity of 94% and a specificity of 95% (Mellby et al., 2018).

Most impressively, however, is the study by Melo et al. that reported an absolute prediction of pancreatic cancer (AUC = 1.0) using glypican-1 on exosomes as identified by MS (Melo et al., 2015). This work has spawned additional interest in the application of exosomes as biomarkers for pancreatic cancer. Carmicheal et al. utilized surface enhanced Raman spectroscopy (SERS) to analyze exosomes derived from pancreatic cancer and normal pancreas cell lines (Carmicheal et al., 2018). A high diagnostic accuracy (90%) was reported for the exosome-based signature in serum.

## Genomic biomarkers

The genetic alterations in pancreatic cancer have been mapped through large-scale genomic studies. Around 60 altered genes in 12 core pathways have been reported for pancreatic cancer (Bailey et al., 2016; Jones et al., 2008). The KRAS oncogene and CDKN2A have been found to be mutated in more than 90% of pancreatic cancers, while the TP53 and SMAD4 genes are mutated in 75% and 55% of the patients, respectively. The PALB2, BRCA1, and BRCA2 genes have been associated with chemotherapy response in a subcategory of pancreatic cancers (Waddell et al., 2015). Altered DNA methylation in mucin genes, including MUC1 and MUC4, is associated with survival in patients with pancreatic cancer (Yokoyama et al., 2016). Klein et al. reported the findings of the largest whole-genome sequencing data analysis of pancreatic cancer to date (Klein et al., 2018). The study included a meta-analysis of 9040 patients and 12,496 controls and found new susceptibility loci for pancreatic cancer. These loci included TNS3, NOC2L, HNF4G, HNF1B, and GRP.

Pancreatic cancer is a complex and heterogeneous disease. Molecular stratification may provide a roadmap for directing treatment and inform on prognosis. Several genomic subclassifications of pancreatic cancer have been put forth. Subclassification based on biological factors suggests three subtypes of pancreatic cancer, including classical, exocrine-like and quasimesenchymal (Collisson et al., 2011). Importantly, tumor subtype correlated with chemotherapy response and clinical outcome. The stroma of pancreatic cancer has also been suggested as a basis for classification. A “basal-like” subtype of stroma was identified and associated with shorter survival. “Normal” and “activated” stroma subtypes were also correlated with clinical outcomes (Moffitt et al., 2015). Bailey et al. suggested four subtypes according to the molecular profile of the tumor, divided into squamous, pancreatic progenitor, and immunogenic, as well as the aberrantly differentiated endocrine–exocrine form (Bailey et al., 2016). However, none of these genomic subclassifications have reached clinical translation and further validation studies are warranted.

## Proteogenomic biomarkers

**Fig. 1:**
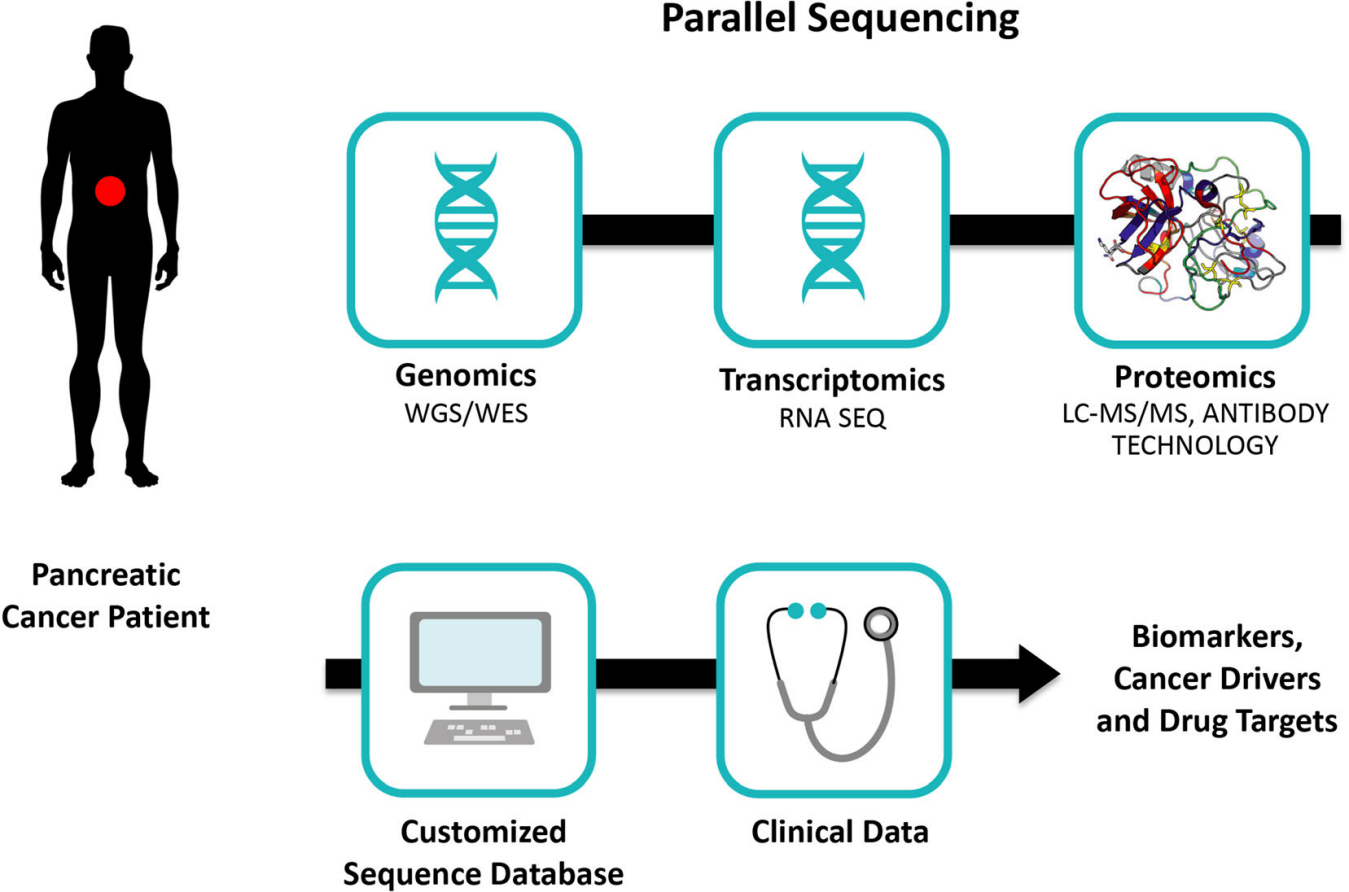
Development strategy for proteogenomic biomarker in pancreatic cancer. LC-MS/MS liquid chromatography tandem mass spectrometry, WES whole-exosome sequencing, WGS whole-genome sequencing

A multidimensional approach based on the integration of proteomic and genomic technology ultimately leads to a better understanding of pancreatic cancer biology and the development of novel types of biomarkers (Fig. 1).

A biomarker strategy combining proteomic and genomic biomarkers was recently reported (Cohen et al., 2017). The study combined CA 19-9, protein biomarkers (OPN, CEA, HGF), and gene mutations (KRAS). A specificity of 99.5% was achieved for diagnosis of pancreatic cancer using plasma samples from patients with pancreatic cancer and healthy controls.

Proteogenomic databases such as PGTools have been developed to integrate proteogenomic data (Nagaraj et al., 2015). Using PGTools, more than 200 new protein coding regions in pancreatic cancer exons were discovered that had previously not been reported by single genetic or proteomic methods. Methods such as PGTools may help to generate peptide sequences from RNA transcripts, classify proteins, identify dysregulated proteins, and report false discovery rates (available at https://omictools.com/pgtools-tool).

## Conclusion

CA 19-9 remains the only approved biomarker for pancreatic cancer by FDA standards, despite extensive research into new markers. This can partially be explained by the extensive validation process necessary to translate biomarker findings into the clinic, including the need for large-scale, multicenter trials that are costly and time consuming. Another challenge is the lack of bioanalytical techniques yielding precise, reproducible analyses of clinical samples with cost-effectiveness for the society at large. However, with the rapidly improving accuracy and throughput of proteomic and genomic analytic instruments, there is a great prospect that proteomic and genomic technologies could be applied in everyday health care.
